# The Case for Vaccination.—From a Pathological Standpoint

**Published:** 1895-12-14

**Authors:** S. Monckton Copeman

**Affiliations:** Medical Inspector Local Government Board, Lecturer on Public Health, Westminster Hospital.


### Medical Progress and Hospital Clinics.

[*The Editor will be glad to receive offers of co-operation and contributions from members of the profession. All letters should be addressed to* The Editor, at the Office, 428, Strand, London, W.C.]

### THE CASE FOR VACCINATION.—FROM A PATHOLOGICAL STANDPOINT.

By S. Monckton Copeman, M.A., M.D. (Cantab), M.R.C.P., Medical Inspector Local Government Board, Lecturer on Public Health, Westminster Hospital.

It is now just one hundred years ago since the process of vaccination as a prophylactic against small-pox was introduced in this country.

This process is probably one of the earliest instances of the employment of a bacteriological inoculation in medicine, although, of course, its true nature was not appreciated at the time of its introduction.

Almost from Jenner's day, however, the value of the practice has by some been impugned, on the plea that inoculation of one disease, "cow-pox," could not be expected to exert any really protective action against the supposed totally different disease of small-pox. And if the thesis of essential difference between these maladies were only capable of scientific demonstration, no doubt such objection would be of considerable weight, seeing that, as far as I am aware, there exists no well-authenticated evidence as to the living virus of one disease being capable, when inoculated into an animal, of affording protection to the system from the effects of inoculation of the virus of a totally different disease, although in such a case the incubation period of the second infection may be somewhat delayed.

That some such thought was probably present to the mind of Jenner may be gathered from the fact of his employing in reference to vaccinia the alternative term variola vaccinæ, or small-pox of the cow. His use of this term would, indeed, appear to indicate his belief that vaccinia was really nothing more nor less than small-pox which had in some way become modified by residence in the organism of the cow.

Even at the present day, however, controversy wages hotly around this point, and consequently the important question still remains as to whether vaccinia is or is not one with small-pox. I prefer in this connection the phrase "one with small-pox," since it matters little from my point of view whether small-pox on its transference from man to the bovine animal becomes "transmuted," as the phrase is, or is merely modified, as some would maintain. If I were informed on good authority that small-pox and vaccinia are descended both of them from a common stock—from an ancestor, for instance, which resembled vaccinia far more than it did small-pox—I should not be disturbed by the proposition; I should perceive, indeed, that the seeming vaccinia obtained in the calf by inoculation of small-pox matter into that animal might, after all, be but a reversion to an antecedent type, and I should be calling to mind a fact of universal experience, namely, that vaccinia (however it may have arisen in the past or is made to appear in the present) exhibits little tendency to "sport" (as, for instance, by manifesting a "generalised" eruption) in the direction of small-pox. I could understand, I mean, a highly differentiated form reverting under artificial conditions to a little differentiated form, though the reverse process readily or frequently repeated *per saltum* would indeed surprise me.

This problem has been attacked from two separate points of views. First, experimental work having for its object production (if that be possible) in the bovine animal, by inoculating it with small-pox lymph, of a disease indistinguishable both in its immediate local and in its subsequent general effects from true vaccinia; and second, microscopic pathology, including, I may add, the testing of bacteria derived from lymph, also on the bovine animal.

With reference to recent experiments on variolation of the calf, it is worthy of note that although different observers have obtained local effects in that animal which in the several calves of a series have varied considerably, the final result has, after greater or less number of removes from calf to calf, been invariably the same, the production, *i.e.*, of a local vesicle indistinguishable by any means at our command, such as the appearance and course thereof, or the lymph derived therefrom, from true vaccinia.

As the result of all the work which has been done in this direction, there can, I think, at the present time be no reasonable doubt as to the possibility of inoculating the calf with variolous matter, with the result of producing thereby after one or more removes a malady which is certainly no longer small-pox, seeing that it has now completely lost, when transferred to the human being, its original property of infectiousness, but which nevertheless is capable of affording protection against the subsequent invasion of true small-pox.

Turning next to the micro-pathology and bacteriology of variolous and of vaccine lymph, it is somewhat curious that although numerous micro-organisms have been isolated from them, which for the most part are mere saprophytes, and to which I have therefore applied the term "extraneous," until recently no organism which could be regarded as specific to these maladies has been discovered. Nearly two years ago, however, Dr. Klein and myself concurrently, though quite independently, demonstrated the fact that in specially stained specimens of vaccine lymph, taken at a period antecedent to full maturity of the vesicles, minute bacilli can be found, often in considerable numbers and in practically pure culture. These bacilli cannot be found, or only with difficulty, in mature lymph, for the reason, probably, that they have by then given place to spores.

These bacilli, and similar ones which can be obtained from small-pox lymph, not only stain with great difficulty, but absolutely refuse to grow on any of the ordinary culture media, and either in presence or absence of air, though this very fact tends to show that they are not of a merely adventitious nature.

During the past year, however, certain experiments which I have devised appear to prove that it is possible to obtain a growth of these bacilli in a particular culture medium, and even to carry on such growth in secondary cultivation.

Calves, also, have been successfully inoculated with such cultures, the resulting vesicular eruption having been carried on through several generations, both in calves and on the human subject.

Of course, these experiments have to be repeated, and are indeed in process of repetition. Should, then, later experience confirm the results obtained thus far, it appears possible that we may eventually be enabled to elaborate a method of specific treatment of individuals actually suffering attack by small-pox somewhat comparable to that discovered by Behring in reference to diphtheria. Should this forecast be justified in the event, some such method would form a fitting complement to the process of preventive vaccination.

